# Are vipers prototypic fear-evoking snakes? A cross-cultural comparison of Somalis and Czechs

**DOI:** 10.3389/fpsyg.2023.1233667

**Published:** 2023-10-19

**Authors:** Daniel Frynta, Hassan Sh Abdirahman Elmi, Markéta Janovcová, Veronika Rudolfová, Iveta Štolhoferová, Kateřina Rexová, David Král, David Sommer, Daniel Alex Berti, Eva Landová, Petra Frýdlová

**Affiliations:** ^1^Department of Zoology, Faculty of Science, Charles University, Prague, Czechia; ^2^Department of Biology, Faculty of Education, Amoud University, Borama, Somalia

**Keywords:** fear, evolutionary psychology, cross-cultural comparison, ophidiophobia, specific phobias

## Abstract

Snakes are known as highly fear-evoking animals, eliciting preferential attention and fast detection in humans. We examined the human fear response to snakes in the context of both current and evolutionary experiences, conducting our research in the cradle of humankind, the Horn of Africa. This region is characterized by the frequent occurrence of various snake species, including deadly venomous viperids (adders) and elapids (cobras and mambas). We conducted experiments in Somaliland and compared the results with data from Czech respondents to address the still unresolved questions: To which extent is human fear of snakes affected by evolutionary or current experience and local culture? Can people of both nationalities recognize venomous snakes as a category, or are they only afraid of certain species that are most dangerous in a given area? Are respondents of both nationalities equally afraid of deadly snakes from both families (Viperidae, Elapidae)? We employed a well-established picture-sorting approach, consisting of 48 snake species belonging to four distinct groups. Our results revealed significant agreement among Somali as well as Czech respondents. We found a highly significant effect of the stimulus on perceived fear in both populations. Vipers appeared to be the most salient stimuli in both populations, as they occupied the highest positions according to the reported level of subjectively perceived fear. The position of vipers strongly contrasts with the fear ranking of deadly venomous elapids, which were in lower positions. Fear scores of vipers were significantly higher in both populations, and their best predictor was the body width of the snake. The evolutionary, cultural, and cognitive aspects of this phenomenon are discussed.

## 1. Introduction

Humans, and presumably other primates, are capable of rapid threat detection through visual perception and specific attention (Kawai, 2019; Kawai and Qiu, 2020), and many theorists consider humans to be predisposed to respond emotionally also to snakes. From this perspective, the fear of snakes has been an evolutionarily relevant reaction to the potential threat of dangerous snakes (Öhman and Mineka, 2003; Isbell, 2006, 2009; Kawai and He, 2016; Landová et al., 2018a). Öhman and Mineka (2001) argue that humans have an evolutionary predisposition to recognize ancestral threats, including snakes. It was proposed that such threats may elicit a complex automatic neurobehavioral response involving early detection through prioritized attention and emotional fear response, followed by an associated rapid behavioral response called the fear module (Öhman and Mineka, 2001, 2003).

However, recent neurobiological studies on non-human primates and those measuring non-invasively human brain activity when participants see snake pictures show that not only subcortical neural systems involving thalamic regions (*superior colliculus* and *pulvinar*, as well as the *amygdala*) responsible for automatic processing are activated, but also cortical neural circuits (mainly involving the right anterior cingulate cortex and medial prefrontal cortex) are specifically engaged during the fear and visual processing of snake stimuli. Moreover, there is high subcortical–cortical connectivity showing that both automatic (LeDoux, 2012), and conscious emotional and cognitive processes are at play (reviewed in Pessoa and Adolphs, 2010; Dinh et al., 2021; see also Nicula, 2020). The amygdala itself was proposed as an important center evolutionary designed to detect and avoid prior interactions with dangerous stimuli, such as snakes (Amaral, 2002; see also LeDoux, 2000, 2012). Bilateral lesions of that nucleus in adult macaques lead to a lack of fear of snakes (Amaral, 2003). The amygdala, together with the pulvinar and superior colliculus, was also activated in response to snake stimuli in tasks involving both implicit (automatic) as well as explicit (goal-directed, experience-influenced) visual and emotional processing of snake stimuli in the human brain (reviewed in Almeida et al., 2015). The metanalysis of fMRI studies reveals that the core fear network comprises the amygdala, pulvinar, and fronto-occipital cortical regions. Both implicit and explicit fear processing share this network, along with the decline of the cerebellum, fusiform gyrus, and middle frontal gyrus. Explicit fear processing activates the pulvinar and the hippocampal gyrus more, which might be related to the context of stimuli presentation and the regulation of fear prominent in explicit fear processing (Tao et al., 2021). Interestingly, Van Le et al. (2013) showed in their single-cell recording study on macaques that some pulvinar neurons are specifically responsive to snake stimuli themselves or to snake stimuli in defensive postures (Van Le et al., 2014).

Apart from the neural substrate for fear processing of snakes as threats (e.g., specific brain activation pattern in ERP studies, Van Strien and Isbell, 2017; Beligiannis et al., 2022; and fMRI studies, Almeida et al., 2015; for details see above), there have also been detailed studies on preferential attention toward snake stimuli (Öhman et al., 2001; Okon-Singer et al., 2011; Langeslag and Van Strien, 2018) that subsequently enables their fast detection (Hayakawa et al., 2011; LoBue and DeLoache, 2011; Soares et al., 2014; Kawai and Qiu, 2020; but see Coelho et al., 2019) and proper recognition (Meno et al., 2013a,b). Various psychological and physiological methods have been used to demonstrate that snakes evoke a significant fear reaction. This includes studies on facial expression (Dimberg and Thunberg, 1998), skin resistance and heart rate (reviewed in Landová et al., 2020), different aspects of the psychophysiological fear reaction (reviewed in Hyde et al., 2019), and subjective evaluation of photographs on elicited fear and disgust emotions (Rádlová et al., 2019, 2020). Interestingly, Morris and Morris (1965) found that not only is the fear of snakes prevalent, but also the attitude toward snakes is negative among both children (27% of them stated that snakes are the animals they dislike the most) and adults (24% of them would not care about snake conservation at all). This negative attitude may contribute to the evaluation of snakes as potential threat (for negative attitude toward snakes, see Prokop et al., 2009; Yorek, 2009; Ballouard et al., 2012; Pandey et al., 2016; but see also Alves et al., 2012 for positive aspects of human attitude toward snakes).

Isbell’s (2006, 2009) snake detection theory (SDT) elaborates on this topic and postulates that during human evolution, snakes represented a substantial selection factor that influenced the evolution of primate vision as well as the human brain. This selection resulted in higher efficiency in detecting this particular type of threat. Isbell (2006, 2009) suggests that venomous snakes in particular played a pivotal role in the later stages of the shared evolution between snakes and monkeys, apes, and human ancestors that shaped the primate visual system and its connections to specific brain regions.

The evolutionary importance of snakes as threat-relevant stimuli is supported by studies demonstrating the innate recognition of snakes as dangerous stimuli in some primates born in captivity (e.g., macaques – Weiss et al., 2015), as well as studies conducted with other animals (birds – Smith, 1977, 1980; geckos – Landová et al., 2016). Strong evidence for pre-existing biases toward snakes comes from human studies with children and infants, which show the existence of non-associatively acquired fear in children (Coelho and Purkis, 2009). Some evolutionary relevant treats, such as snakes, can also become objects of “privileged” learning, as showed in some developmental studies (reviewed in LoBue and Rakison, 2013). LoBue and DeLoache (2008) showed that pre-school children (ages 3–5) exhibit shorter detection time in visual search tasks when identifying snake images, even among morphologically similar caterpillars. Both American (ages 2–5) and Indian children (ages 3–8, from rural and urban areas) discriminate snake and lizard pictures more quickly in similar visual search tasks and have shorter reaction times to snake stimuli (Penkunas and Coss, 2013a,b). Even very young children (8–14 months old) turned more quickly to the threatening stimuli, which included snakes and angry faces than to the neutral ones (LoBue and DeLoache, 2010; see Bertels et al., 2018 for similar results). In comparison with frogs and caterpillars, snakes also generated a specific and higher pattern of brain activity in the occipital region in 7–10 months old infants (Bertels et al., 2020). In their subsequent EEG study, Bertels et al. (2023) showed that both color as well as greyscale pictures of snakes evoked a specific pattern of activation and that this snake-specific response strengthens with age, likely reflecting the refinement of the developing visual system (Bertels et al., 2023).

The process of responding to the threats that snakes, whether in general or specific species in certain situation, may represent, is a complex process. It involves snake detection, recognition (or recognition of a particular snake category), accompanied by subjectively perceived emotions, and decision-making when it is necessary to choose an appropriate reaction toward the threat that the particular snake stimulus represents. The question is how non-human primates, as well as humans, deal with assessing snake appearance and what morphological traits contribute to subjectively perceived fear (level of threat) as well as to the detection and recognition of snakes.

Among reptiles, snakes possess a distinctive morphotype that contributes to snake recognition (Janovcová et al., 2019). In particular, one study reports that an important characteristic of threat detection is the curvilinear body shape itself, especially if the participants know that there might be a snake or when they are primed by another fear stimulus (LoBue, 2014). However, the curvilinear shape of snakes still evokes a stronger brain response than the curvilinear shape of the worms (as shown in an ERP study, Van Strien et al., 2016). When respondents subjectively evaluated the fear elicited by picture stimuli covering the full scope of morphological variability among snake subfamilies, the most salient traits of the snake were body width and head length (Rádlová et al., 2019). When respondents evaluated live kingsnakes (*Lampropeltis*), the body size and the black color were the salient stimuli (Landová et al., 2012). The typical snake scales, as well as the different patterns that scales form on the snake’s body, are important features for early selective visual processing, as shown in studies using the event-related potentials in humans (Van Strien and Isbell, 2017; see also Kawai, 2019). Even very young children (7–15 months old) poked more at plastic cylinders with snake scale patterns, and even younger children (5 months old) gazed longer at them compared to those with geometric shapes or plain colors (Coss and Charles, 2021). Ethological studies on non-human primates also report that vervet monkeys (*Chlorocebus pygerythrus*) are able to detect and recognize snakes based on small pieces of snakeskin only (Isbell and Etting, 2017). Similarly, Colombian white-faced capuchin monkeys (*Cebus capuchinus*) respond more intensively with antipredator behavior if scales were present on a snake model (Meno et al., 2013a). Interestingly, the color of snake stimuli (except for a minor effect of color contrast) did not facilitate a specific pattern of brain activation in very young infants (Bertels et al., 2023). Even the aposematic coloration of some snakes does not increase the fear of snakes (Prokop et al., 2018).

We suggest that the source of natural selection (being endangered by venomous snakes) that contributed to the rapid detection of snakes may persist in modern times (at least in some areas, see below). Many modern human populations have current as well as evolutionary experience with different types of snakes; some of these snakes represented danger mainly in the evolutionary past, while others (namely venomous snakes, reviewed in Landová et al., 2021) continue to pose a serious threat to people even today (Akani et al., 2013; Pandey et al., 2016; Onyishi et al., 2021; Staňková et al., 2021). As the evaluation of the threat that animals may represent encompasses implicit automatic reactions, as well as long-term goal-directed cognitive and emotional evaluations labeled as explicit processes (Effting et al., 2016), we can assume that both processes collaborate in the subjective evaluation of fear elicited by particular animal species. This level of subjectively perceived fear may be related to overall decision-making about the potential level of threat, and it is also connected with the subsequent behavioral reaction (see Landová et al., 2023, for how the subjective fear evaluation of pictures and individual fearfulness are related to overall brain activity). This raises the question of whether modern humans can distinguish venomous snakes from non-venomous snakes based on the degree of subjectively perceived fear and how this degree of subjectively perceived fear is affected by the risk that venomous snakes pose today and in the evolutionary past (Bertels et al., 2023).

There are some pieces of evidence indicating that people are able to recognize dangerous venomous snakes. In our previous papers, we selected from a wider variability of snake species those that evoked high fear (while evoking low disgust) when presented to the participants in the picture. Many of these species were vipers (Rádlová et al., 2019, 2020). Subsequently, these fear-evoking snakes elicited stronger psychophysiological emotional reactions measured as a change in skin resistance and heart rate (Landová et al., 2020). In a cross-cultural comparison between the Czech population (where the risk of snakebite is low) and the Azerbaijani population (where the risk of envenomation is relatively high), we found that both populations fear the cobra (but only when presented in a threatening posture) and vipers the most. Interestingly, there was a high cross-cultural agreement on the subjective emotional evaluation of pictures, even though the attitude toward snakes was generally more negative in Azerbaijan. However, only one species of cobra was included in this set of picture stimuli, hence the potential discrimination between cobras, vipers and non-venomous snakes could not have been tested (Landová et al., 2018a).

From a cognitive perspective, the task of ranking multiple snake species according to the level of fear they evoke becomes a categorization task, especially when some of the snakes are or were dangerous to the investigated population in their evolutionary past while others are (were) not. Categorization of emotionally relevant stimuli is a cognitive process (Meriau et al., 2006; Brosch et al., 2010; Wieser and Brosch, 2012; Harnad, 2017), in which both the perceptual similarity of the objects and emotional sensitivity to the feared objects play important roles (Landová et al., 2021). This cognitive process involves transforming a real object that triggers emotions into a percept, representing the accessible subjective experience associated with the activation of a certain category in the mind (Brosch et al., 2010). Furthermore, this process influences extended attention toward evolutionarily relevant threatening stimuli (Grassini et al., 2019).

This cognitive process is influenced by the evolutionary past, the current risk represented by the snakes in the respective countries, and the local culture. In cross-cultural comparisons, these three major factors may influence both investigated populations similarly (such as the evolutionary past) or their effect may substantially differ (such as the risk of envenomation that could correlate with the abundance of deadly venomous species or the various cultural backgrounds). Each of these key factors is applicable to modern humans in general, and their specific effects on Somali and Czech populations need to be introduced. Firstly, we will delve into the evolutionary history of human ancestors, beginning with the earliest hominids. We will focus on the two regions of interest relevant to this paper (i.e., the Horn of Africa and Central Europe) in order to establish the extent of shared evolutionary history. We will also introduce the evolutionary history of venomous snakes in Africa and the regions through which current Europeans migrated with the intent of establishing the approximate length of sympatry between respective human populations and region-relevant fauna of venomous snakes. Secondly, we will estimate the current risk that the venomous snakes represent in Somaliland and the Czech Republic. Thirdly, we will describe the attitudes toward snakes specific to Somali and Czech cultures based on unstructured interviews with locals as well as our own experiences in these locations.

### 1.1. Primate evolution in Africa

Phylogeographic analyses suggest that the common ancestor of Hominoidea (gibbons, great apes, and humans) and Cercopithecoidea (Old World monkeys) was living in the Asian continent (Springer et al., 2012). The divergence time between these two superfamilies of Old-World primates (Catarrhini) was estimated to be the Oligocene period (Springer et al., 2012), during which the first fossils of the monkey *Aegyptopithecus zeuxis* are reported from the Egyptian oasis Fayum (Simons et al., 2007). This fossil is currently interpreted as stem catarrhine (Urciuoli et al., 2021). In the Miocene, multiple ape species were distributed across Africa, Arabia, S and SE Asia, and even Europe (Almécija et al., 2021). Some authors have emphasized the hominin affinities of certain European Miocene hominids (Begun et al., 2012; Fuss et al., 2017; Kirscher et al., 2021), suggesting a potential role for Europe and the Near East in human evolution during that period. Nevertheless, hominines, i.e., gorillas and chimpanzees, ~7–6 million year-old fossils of *Sahelanthropus* and *Orrorin*, ~4–3 million year-old australopith fossils, and early *Homo* are found exclusively on the African continent (Senut et al., 2001; Almécija et al., 2021). Thus, it is more parsimonious to consider that human evolution took place there. The split between gorillas and the human-chimpanzee clade is currently estimated to be ~11 million years ago (Langergraber et al., 2012). This provides the shortest estimate of the time our ancestors spent in the African environment. For this long period, they were exposed to the pressure of local snakes. Nevertheless, for a considerable portion of this time, our ancestors, including the last common ancestor with chimpanzees (~9.3–6.5 my, Moorjani et al., 2016), inhabited forest habitats rather than savannas (Andrews, 2020).

### 1.2. Evolution of human ancestors in the African horn

African continent, namely its eastern part including the African Horn is usually declared as the cradle of humankind. The African Horn and adjacent parts of East Africa belong to the regions with the best-documented fossil record of early hominines including australopithecines, as well as *Homo ergaster*/*erectus* (Clark et al., 1994; Abbate et al., 1998; Asfaw et al., 2002; Profico et al., 2016; Gallotti and Mussi, 2017) and ancestor of modern humans usually referred to as *H. heidelbergensis*/*H. rhodesiensis* (e.g., locality Bodo, 600 thousand years ago; Conroy et al., 2000; Rightmire, 2009). This also concerns early modern humans. Fossils of the earliest anatomically modern humans are exclusively of African origin (Stringer, 2016). There are fossil records, e.g., from Ethiopian Awash (locality Herto, 154–160 thousand years ago, White et al., 2003,) and Omo (locality Kibish, 195 thousand years ago, McDougall et al., 2005). Some scholars are placing even older Mid-Pleistocene fossils from various African sites to this lineage (Gibbons, 2017), this especially concerns those of Moroccan Jebel Irhoud (~300 thousand years ago, Hublin et al., 2017; Richter et al., 2017).

Taken together, this suggests a continuous presence of human ancestors in the African Horn and more generally speaking the Ethiopian and East African Rift Valley, which clearly suggests that human evolution occurred in this region. This means that animal species present during this evolutionary history in this landscape had a chance to interact extensively with human ancestors. However, the original hypotheses suggesting that savannas east of the Rift Valley represent the only area of human evolution were already falsified by the presence of multiple fossils outside this region in other parts of Africa (Hublin et al., 2017; Richter et al., 2017). Moreover, genetic data revealed multiple admixture events within Africa during the Mid-Pleistocene period, e.g., ghost archaic introgression in African populations (Durvasula and Sankararaman, 2020) and human immigration flow from South Africa to East Africa ~70 thousand years ago (Rito et al., 2013, 2019). Archeologic and paleoclimatic data suggest that human populations during the Mid-Pleistocene were at least strongly divided temporarily by environmental barriers (Saharan region – Scerri et al., 2014, Eastern versus Southern Africa – Rito et al., 2019).

The region of the African Horn also represents the suggested source area for colonization of the Arabian Peninsula and Asia by anatomically modern humans via the Bab Al-Mandab (for Out of Africa scenarios, see Groucutt et al., 2015).

### 1.3. Sources of human populations in Central Europe

After crossing the border of the African continent (>50,000 bp, Bergström et al., 2021), modern humans immigrated to the Arabian Peninsula and the Middle East area, where they hybridized with the Neanderthals (*Homo sapiens neanderthalensis*). Then they rapidly colonized South Asia and Sahul (New Guinea and Australia). The first wave of modern humans reached Central Europe ~45, 000 years bp. However, genetic data clearly showed that current Europeans are not descendants of these early Palaeolithics (Prüfer et al., 2021). European populations are a mixture of at least three source populations: (1) The Western hunter-gatherers, descendants of the second wave of European Palaeolithics, (2) the Western early farmers (Anatolian neolithics), and (3) the Ancient Euro-Asians (Lazaridis et al., 2014, 2016). In the contemporary populations of Central Europe, the third component is dominant (Haak et al., 2015). It can be attributed to massive immigration from the Russian steppes around 4,800 years bp that substantially changed the genetic composition of the human populations in Central Europe (Olalde et al., 2018; Papac et al., 2021).

### 1.4. Venomous snakes of Africa

Many snakes and some lizards belonging to the clade Toxicofera (Reptilia: Squamata) produce toxins (Fry et al., 2009, 2012; Dobson, 2022), but truly venomous snakes possess also specialized fangs. Besides vipers (Viperidae) and rattlesnakes (Crotalidae) with specialized solenoglyphous fangs, there are two families – Atractaspididae and Elapidae – that evolved proteroglyphous fangs (Portillo et al., 2019; Westeen et al., 2020). Moreover, there also are a few highly toxic colubrids, like African boomslangs of the genus *Dispholidus*, which are equipped with fangs morphologically closely resembling those of elapids (Westeen et al., 2020). Except for pit vipers (Crotalinae), which are distributed solely in Asia and America, all the other families of venomous snakes are represented in Africa and therefore are relevant to potential interactions with human ancestors.

### 1.5. Phylogeography and evolutionary history of African venomous snakes

Viperids diversified during the Eocene/Oligocene boundary, but their ancestral area is not well-resolved (Asia/Arabia/Africa). The earliest split separates the purely African genus *Causus* from the remaining viperids. Next Early Oligocene split separates Asian and/or European vipers from the Afro/Arabian clade consisting of five genera that split in the Oligocene. While the clade comprising genera *Proatheris*, *Atheris,* and *Bitis* is exclusively African, the other one, consisting of *Cerastes* and *Echis* is distributed in both Africa and Arabia/Asia (for details see Pook et al., 2009; Šmíd and Tolley, 2019). Thus, it is still uncertain whether the genus *Echis* evolved in Africa or Arabia and thus, we are unable to set precise dating of its evolutionary interactions with human ancestors living in the African continent. Nevertheless, *Echis* was likely present in Africa from the Miocene, most probably from the Middle Miocene period (~16 million years, Šmíd and Tolley, 2019). In contrast to *Echis*, the long-term continual presence in Africa, biogeography and ecology of its diversification are well-documented in the case of the genus *Bitis* (Barlow et al., 2019). These vipers are currently distributed across Sub-Saharan Africa (+Morocco and S Arabia) from lowlands to high mountains and from wetlands to xeric habitats. The position of the giant species of *Bitis* on the phylogenetic tree suggests that large-bodied forms possessing high amounts of venom are not of recent origin.

Elapidae belongs to the clade Elapoidea which evolved and radiated in Africa during the late Eocene and comprises also families Lamprophiidae, Pseudoxyrhophiidae, Atractaspididae, and Psammophiidae (Kelly et al., 2009; Zaher et al., 2019). Elapids radiated initially in Asia during Oligocene. African mambas represent a sister clade of Asian King cobras (*Ophiophagus*). African cobras form a distinct clade also including some Asian species. Both these clades, i.e., mambas and cobras, evolved in Africa during the Miocene period (ca 20 million years ago, Kelly et al., 2009).

Atractaspididae is of African origin. The Guinean-Congo region is probably an ancestral area of this clade that further radiated, namely in the Zambezian region, during the Oligocene and Miocene periods (Portillo et al., 2018, 2019).

We can conclude that venomous snakes (solenoglyphous vipers, and proteroglyphous elapids and atractaspidids) currently inhabiting the African continent and representing a risk for humans have been already present in Africa for the last 30–20 million years. Also, the remaining principal clades of African caenophidian snakes have a long history on this continent as exemplified by the genus *Telescopus* of the family Colubridae (Šmíd et al., 2019) and families belonging to Elapoidea.

### 1.6. History of interactions between the Europeans and venomous snakes

On the way from Africa to Central Europe, ancestors of the Europeans were exposed to multiple viper species. While the snake fauna of the Arabian Peninsula and adjacent areas resembles that of North-Eastern Africa, the Middle East has its own vipers of the genera *Macrovipera*, *Montivipera*, *Daboia,* and *Vipera*. Especially, the Levant viper (*Macrovipera lebetinus*) is large-sized, deadly venomous, and widely distributed across the region. The Volga River region of Russia which represents the source area of the Eneolithic migration wave to Europe (see above) is inhabited by the Karaganda pit viper (*Gloydius caraganus*). It is a small-sized moderately venomous species belonging to the genus *Gloydius* of Central Asian origin that diverged about 2.5 million years ago (Asadi et al., 2019). Smaller insectivorous species of adders from the *Vipera ursini-renardi* complex (Mizsei et al., 2017) resemble other viper species, but due to their smaller size, they are much less venomous. They have a highly fragmented distribution, ranging from Eastern France to Western China (Nilson and Andrén, 2001). Fossil records are known from the lower Pleistocene (0.8–1.8 million years) from the Czech Republic (Szyndlar, 1991). This *ursini-renardi* complex was not of interest because it did not represent a risk for humans. The adder or Northern viper (*Vipera berus*) is the only venomous snake currently reported from the central and northern parts of Europe. The geographic range of this adder is the largest among snake species, it extends from Western Europe to Siberia and the Far East, and even crosses the Polar Circle in the North. All northern populations of the adder across Eurasia are genetically similar forming a single clade (Cui et al., 2016). The evolutionary roots of the species are in Southern Europe as suggested by the presence of the distinct mitochondrial clades in the Balkan Peninsula and Northern Italy, and the occurrence of related species of the genus *Vipera* there (Ursenbacher et al., 2006). Recently, palaeontological data confirmed the presence of the adder in Central Europe during the late Pleistocene glacial period (Ivanov and Čerňanský, 2017). Thus, the adder has been present there since the appearance of the first populations of modern humans.

### 1.7. Conclusion on the history of interactions between venomous snakes and Somalis and Czechs

It seems certain that the genus *Homo* evolved in Africa. Based on the split between gorillas and human-chimpanzee clade, humans and human ancestors have inhabited the African continent for at least the last 11 million years. During this whole time, highly venomous snakes including African mambas, cobras, and vipers of the genera *Bitis* and *Echis* were already present on the continent as these clades came to Africa no later than 16 million years ago (Barlow et al., 2019; Kelly et al., 2009; Šmíd and Tolley, 2019). Thus, at least 11 million years long evolutionary interaction between human ancestors and these groups of venomous snakes can be expected. There is no reason to suspect that Somali ancestors ever left the African continent (or adjacent Arabian Peninsula) thus their evolutionary experience with investigated snake stimuli is uninterrupted.

Contrarily, Czech ancestors at some point left the African continent. While the precise date is currently not known, it was probably between 100,000 and 50,000 years ago (Groucutt et al., 2015; Bergström et al., 2021). It is assumed that they spent a significant amount of time in the wider Middle East area before migrating further north and finally arriving in Central Europe by different routes, mainly through the Russian steppe around 5,000 years ago. The ophiofauna of the passed-through regions has been increasingly less diverse. While there are still several highly venomous species of vipers and cobras in the Middle East, only “moderately” venomous viper and pit vipers are present in Siberia and the Russian Steppes. Finally, only one “moderately” venomous viperid (*Vipera berus*) and no elapid species can be found in Central Europe. It can be concluded that Czech historical experience with vipers, in general, is also uninterrupted, although, during the last several thousand years, the viper diversity and objective fear relevance significantly decreased. Since leaving the Afro-Arabian region, Czech ancestors have not been exposed to elapids furthermore. However, some animal studies show that caution and antipredator behavior toward predators may persist even after thousands of years of relaxed natural selection due to the absence of their former predators (Coss, 1991, 1999; Blumstein, 2006).

### 1.8. Current significance of venomous snakes as a selective pressure upon human populations

Snake bites have been considered only a marginal source of human mortality until recently. The annual number of envenomations and deaths in the entire Sub-Saharan Africa was estimated to be 314,078 and 7,331, respectively (Chippaux, 2011). Recent update reports 268,471 cases of envenomation, 12,290 deaths, and 14,766 amputations (Halilu et al., 2019). This results in a health burden comparable to other neglected tropical diseases (Habib et al., 2015). The real snake bite-envenomation burden is probably underestimated due to incomplete reporting. Moreover, the mortality of patients who do not attend modern health centers is roughly four times higher (Chippaux, 2011). In recent years, studies reporting the incidence of snake bites and consequent human mortality were performed also in the Philippine Agta (Headland and Greene, 2011), an indigenous community in Southeast Nigeria (Onyishi et al., 2021), or the countries of African Horn (e.g., Aga et al., 2014; Fekadu, 2016). Unfortunately, most snake bites are carried out during the night and relevant determination of the snake is missing in most cases (e.g., Nhachi and Kasilo, 1994).

### 1.9. Deadly venomous snakes in the African horn and Central Europe

**Figure 1:**
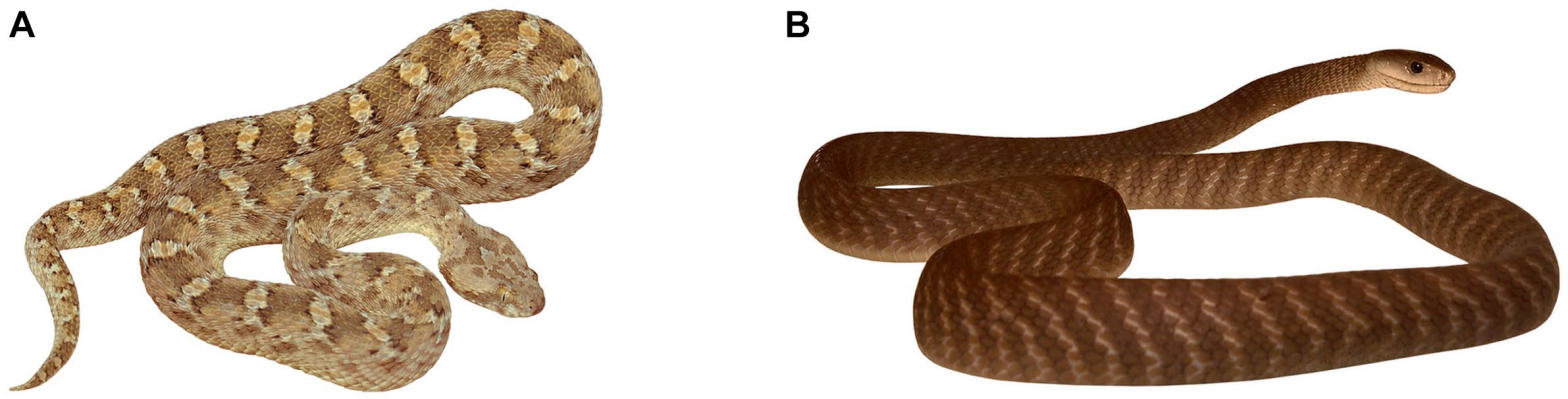
The most dangerous snakes of the African Horn. **(A)** Northeast African carpet viper *Echis pyramidum*, authors of the original photo Daniel Frynta and Petra Frýdlová. **(B)** Black mamba *Dendroaspis polylepis*, author of the photo Martin Smrček. These photos were used with the permission of the authors.

In Somaliland, both vipers and elapids represent a considerable source of envenomation and mortality. Northeast African carpet viper (*Echis pyramidum;* Figure 1A) and puff adder (*Bitis arietans*) are deadly venomous viperids and they both belong to common snakes in Somaliland (Lanza, 1990). Although the range of the Egyptian saw-scaled viper is geographically restricted to lowland semi-deserts and dry-savannas, it is locally highly abundant in certain areas of Somaliland. All elapid species living in the territory of Somaliland are highly venomous (Ainsworth et al., 2018), especially the black mamba (*Dendroaspis polylepis;* Figure 1B), but also the Egyptian cobra (*Naja haje*), the red spitting cobra (*N. pallida*), and the giant spitting cobra (*N. ashei*).

The European common viper (*Vipera berus*) is the only venomous snake reported from the central and northern parts of Europe. In the Czech Republic, cases of envenomation are rare, and fatalities have not been reported for at least two decades (Valenta, 2010). The current risk of envenomation and subsequent injury or death is still substantial in Somaliland but negligible in the Czech Republic.

### 1.10. Cultural attitude toward snakes in Somaliland and the Czech Republic

Attitudes toward snakes differ between the two studied populations. Preliminary interviews with Somali pastoralists and students revealed that their attitude toward snakes is very negative. They relate negatively to all snakes and, for example, have a hard time believing that some people may like snakes and consider them beautiful. Their attitude toward animals in general is strongly driven by their potential use and snakes are considered useless at best. They do not hesitate to deliberately kill a snake in case of an accidental encounter. Most participants were able to recognize a few snake species, usually the most dangerous or the most common. For example, the Northeast African carpet viper is small and difficult to notice but also abundant and highly venomous, making it probably the most lethal snake of Somaliland. This species is well known among the people and Somali participants frequently recognize it among images shown to them.

The attitude of the Czech population is rather ambivalent. On the one hand, children are already taught in elementary school what a viper looks like and that it is venomous. On the other hand, all snakes are protected by Czech law and their value for the ecosystem is also taught. The predominant reaction is to keep a distance from the snake due to fear. Although most people reported the experience of encountering a snake in nature, very few people have ever killed or seen someone kill a snake. In cases when they reported this experience, it mostly happened in the context of traffic or other accidents (Landová et al., 2018a). There is a small but not insignificant number of Czechs that keep snakes as their pets. Moreover, a large part of the population can appreciate the beauty of at least some snakes (Janovcová et al., 2019).

### 1.11. Aims and predictions

The aims of this paper are as follows: (1) To assess whether participants exhibit more subjectively perceived fear of deadly venomous snakes compared to non-venomous or slightly venomous ones. As there are two clearly distinct categories of deadly venomous snakes in Africa and adjacent Eurasia, the vipers and the elapids, we included both these groups. Non-venomous snakes were also represented by two categories: the sand boas and the non-elapid Elapoidea + Colubroidea. If participants do not differentiate between the snakes in terms of subjectively perceived fear elicited by the stimuli (null hypothesis), there should be a low agreement among participants regarding which snake is subjectively perceived as the most fear-evoking. Conversely, if there is high congruence, we will analyze whether the respondent’s subjective fear is associated with particular species or group(s), and further, whether any morphological features of the snakes are correlated with the propensity of elicited fear. (2) To compare the fear ranking of Somali and Czech participants. Cross-cultural agreement in the subjective fear evaluation of snakes would provide additional support for the findings of developmental studies (see above), suggest strongly that the mechanisms regulating fear are innate. Specifically, such a result might reflect an innate modulation of higher cognition resulting from exposure to snakes during human evolutionary history. Conversely, an opposite result would favour the role of current experience and/or local culture.

## 2. Materials and methods

### 2.1. The Somali respondents

We performed the research at the campus of Amoud University in Borama. Most of the respondents were undergraduate students of various fields who agreed to voluntarily participate in the experiment. The students came not only from the Borama region itself but also from other provinces of Somaliland and adjacent Somali-speaking countries. A total of 155 Somali respondents finished the task (for the data see Supplementary Table S1). They were 122 men and 33 women. The mean age was 21.95 years (median = 22, range 18–27).

### 2.2. The Czech respondents

We gathered the respondents among students, mostly of technical and other non-zoological disciplines. Although all students and staff were tested at universities in the capital city of Prague, they come from different parts of the Czech Republic, from smaller towns and villages. They were 90 men and 54 women. The mean age was 19.65 years (median = 19, range 18–42).

### 2.3. The stimuli

We selected 48 snake species belonging to four distinct groups, each represented by 12 species/subspecies. The first two groups comprised highly venomous snakes: (A) The vipers of the family Viperidae belonging to the genera *Bitis*, *Cerastes*, *Echis*, *Macrovipera*, *Montivipera* and *Vipera*. Two representatives of each viperid genus were included. (C) Cobras and mambas of the family Elapidae including 9 cobras of the genus *Naja*, as well as the Cape coral cobra (*Aspidelaps lubricus*), and the black mamba *(Dendroaspis polylepis).* Moreover, we included into this category boomslang (*Dispholidus typus*), a highly venomous colubrid morphologically resembling the elapids. The remaining two categories comprised non-venomous (or only mildly venomous) snakes. (B) Sand-boas of the genus *Eryx* (11 species) and rubber boa (*Charina bottae*) represented a category of fossorial boids, while the remaining species (further referred to as “colubrids“) belong to (D) families Colubridae (*Crotaphopeltis, Philothamnus*, *Telescopus,* two species of *Platyceps* and *Dasypeltis*), Psammophiidae (two species of *Psammophis*) and Lamprophiidae (two species of *Boaedon* and *Limaformosa capensis*). For a complete list of the species and scientific names of the stimuli see Supplementary Table S2.

### 2.4. Stimuli preparation

**Figure 2:**
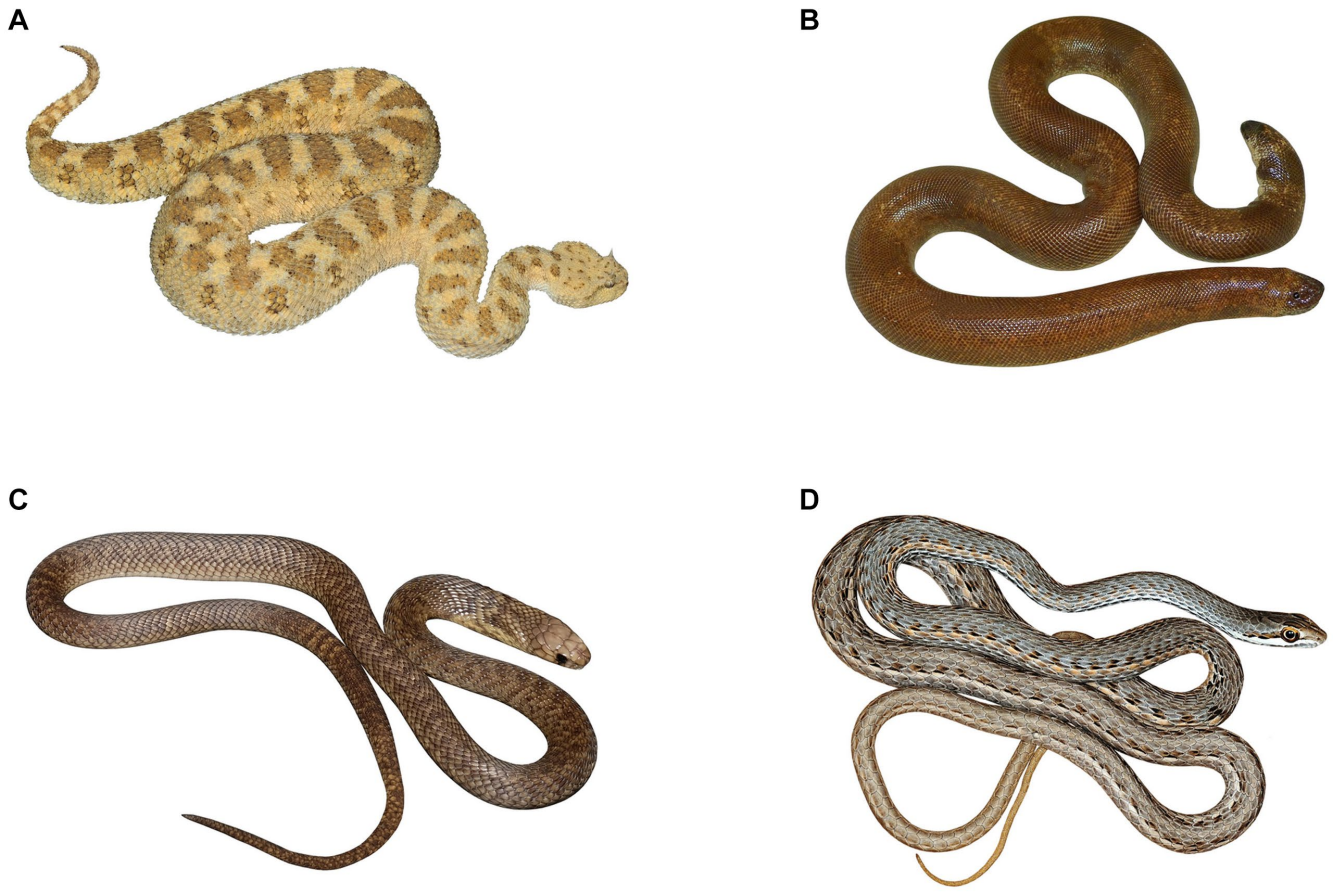
The example of experimental stimuli. The set of photos of snakes (48 stimuli) contains four distinct categories, differing in the level of danger and body shape. Category **(A)** – vipers (desert horned viper *Cerastes cerastes*, authors of the original photo Daniel Frynta a Petra Frýdlová), category **(B)** – sand boas (red sand boa *Eryx johnii*, author of the photo Markéta Janovcová), category **(C)** – elapids (Egyptian cobra *Naja haje*, authors of the photo Daniel Frynta a Petra Frýdlová) and category **(D)** – “colubrids” (Tanganyika sand snake *Psammophis tanganicus*, author of the photo Tomáš Mazuch). These photos were used with the permission of the authors.

For each species from the list, we selected a relevant picture. The source photographs were adopted from the authors’ archives and archives of Tomáš Mazuch; half of the species were from online sources (see Supplementary Table S2). To avoid possible effects of the background and size of the stimulus on rankings, we digitally placed the animals on a white background. We also resized them so that the pictured animals were of a similar size. For the example of experimental stimuli, see Figure 2. Then we printed the final stimuli as photographs 100 × 150 mm in size. We previously showed that fear evaluation of standardized pictures highly correlates with that of live animals (Landová et al., 2012).

### 2.5. Extraction of morphological characteristics

To analyze the shapes of snakes that evoke the greatest fear in humans, we extracted 9 morphological characteristics. Using Image Tool (Wilcox et al., 2002), the measured traits were total body length, body width, head length and width, neck width, tail width and eye diameter, all traits are in millimeters and were not further modified for analysis. Additional characteristics were extracted using the Image J (Rasband, 2016) program, specifically perimeter and body area (silhouette), both measured in pixels. The body area was square-root transformed for analysis, the perimeter was not modified. All morphological characteristics (Supplementary Table S3) were measured on standardized photos of the stimuli because we were interested in how people perceived the depicted snakes. For this reason, the real body dimensions of the included species were not used.

### 2.6. The task

At the beginning of the task, a respondent was standing in front of a well-lit table. We provided him/her with a set of 48 pictures packed in random order. We asked the respondents to imagine the pictures as real animals. Then we asked him/her to place all stimuli on the table in a random assemblage. This sometimes required assistance to ensure that the stimuli were oriented properly, i.e., the top margins of the stimuli were oriented toward the top of the table. The task was to pick up the picture of an animal that was the most fear-evoking, then to pick up the second most fear-evoking one, until he/she picked up the least fear-evoking stimulus on the table. In the end, the respondent had a whole pack of pictures in his/her hand. Finally, each respondent was asked for age information and their gender was recorded. The entire task took most respondents approximately 15 min. The picture order in the pack was then coded from 1 (the most fearful one) to 48 (the least fearful one), further referred to as ranks.

We previously applied this rank-order method in multiple studies evaluating either the beauty of animal stimuli (e.g., Marešová et al., 2009a,b; Frynta et al., 2011, 2013; Lišková and Frynta, 2013; Landová et al., 2018b) or emotions evoked by animals (e.g., Rádlová et al., 2019). It maximizes the informative content of the respondents’ judgment by covering the full ordination scale (Lišková et al., 2015). We repeatedly demonstrated that mean ranks were highly correlated to scores produced by the 5- or 7-point Likert scale (e.g., Frynta et al., 2010; Rádlová et al., 2020).

We are confident that we are measuring subjectively perceived fear by this method. In previous research, we established a correspondence between the evaluation of the level of subjectively perceived fear elicited by pictures of snakes or spiders, psychophysiological reactions (such as skin resistance and heart rate), and the intensity of brain activity that we measured in fMRI. The majority of these parameters related to the level of subjectively perceived fear, elicited by snake or spider photographs, also closely aligned with the behavioral parameters measured in the behavioral approach test (Landová et al., 2020, 2023). Furthermore, in non-human primates, realistic photographs (in size and color) placed in the natural context, stimulate anti-predator behavior in capuchin monkeys (Meno et al., 2013a,b). Therefore, using photos of snakes should yield similar results as presenting actual snakes. Hence, this method can serve as an efficient protocol to initiate decision-making in perceivers regarding any particular snake species based on their subjectively experienced emotions.

### 2.7. Ethical note

The study was approved by the Institutional Review Board of Charles University, Faculty of Science (approval no. 2019/2011, granted on 27 March 2019) and Amoud University, School of Postgraduate Studies & Research (approval no. AU/AA/0012/2021, granted on 7 January 2021).

### 2.8. Data analysis

As the data were ranks, we adopted non-parametric statistics which are appropriate for analyzing these datasets. In order to quantify agreement among the respondents, we computed Kendall’s coefficient W, as implemented in the package irr (Gamer et al., 2012). To compare the mean ranks of individual stimuli we first calculated the Friedman test enabling us to prove the significant effect of species. Then we employed the *post-hoc* Friedman-Neményi test permitting reliable multiple comparisons among the stimuli. The output was a matrix of *p*-values. These tests are available in PMCME and PMCMRplus packages (Pohlert, 2014). In addition, we employed RDA (Redundancy Analysis), as implemented in the package vegan (Oksanen et al., 2020), to assess the variance in the original data which is constrained by country, gender, age and their interactions. All these calculations we carried out in R-environment (R Development Core Team, 2012).

We calculated the means and median values of ranks for each stimulus/species. The values were further analyzed. To obtain more intuitive values increasing with fear (not decreasing as original ranks and its means) and ranging from 0 to 100, we calculated the following index: Fear = 100–(100 * (median rank – 1)/(the number of examined stimuli – 1)).

To compare fear elicited by different groups (categories) of snakes, we ran the Kruskall–Wallis test with *post-hoc* comparisons. We also employed a cluster analysis to uncover groups of stimuli treated by the respondents in a correlated way. We extracted the dissimilarity matrix from the ranking dataset (1-Pearson’s *r*) and applied Ward’s method of clustering. These calculations were performed in Statistica 9.1 (StatsSoft Inc, 2010).

## 3. Results

### 3.1. Agreement among the respondents

We found significant agreement among 155 Somali respondents as well as among the 144 Czech ones. Kendall‘s coefficients of concordance (Wt) were 0.131 (chi-square_(47)_ = 951, P < <0.0001) and 0.269 (chi-square_(47)_ = 1818, *p* < <0.0001; men: *n* = 90, Wt = 0.280; women: *n* = 54, Wt = 0.263), respectively. The descriptive statistics for each stimulus are given in Supplementary Table S4.

The RDA with permutation test revealed that the effects of gender and age on the evaluation of the stimuli are negligible. The best model (AIC = 2667.74) includes the country (Somali vs. Czech) as the only factor constraining 4.03% of the variation in the entire data set (anova: *F*_(1,297)_ = 12.48, *p* < 0.001).

### 3.2. *Post-hoc* comparisons among stimuli species

Friedman tests proved that the effect of the stimulus on perceived fear was highly significant in both Somali and Czech datasets (*p* < < 0.0001). Thus, we calculated Friedman-Neményi comparisons among stimuli (= snake species). Out of 1,128 *post-hoc* comparisons among the stimuli, 458 (40.6%) and 610 (54.1%) were significant in the Somali and the Czech datasets, respectively (*p* < 0.05, for the matrices of *p*-values, see Supplementary Tables S5, S6).

### 3.3. The patterns of elicited fear among the stimuli

In the Somali sample, vipers appeared to be the most salient stimuli. They occupied all the first eight as well as the 10th, 11th, 17th, and 18th positions according to subjective fear. Thus, 10 of 12 viperids were placed above the upper quartile. This strongly contrasts with the fear ranking of elapids. The only elapid placed above the upper quartile was *Naja mosambica* on the 9th position. Interestingly, 8 out of 12 species below the lower quartile were deadly venomous elapids, the black mamba (*Dendroaspis polylepis*) being at the bottom of the fear ranking (Figure 1B; Supplementary Table S4).

In the Czech sample, the privileged position of the vipers is even more pronounced, they occupy all 12 places above the upper quartile. They are followed by the sand boas occupying eight of the remaining 12 positions above the median fear (i.e., in between the upper quartile and median). Elapids, except of *Aspidelaps lubricus*, *Naja pallida* and *N. mosambica*, are below the median fear value.

### 3.4. Comparing categories of examined snakes

**Figure 3:**
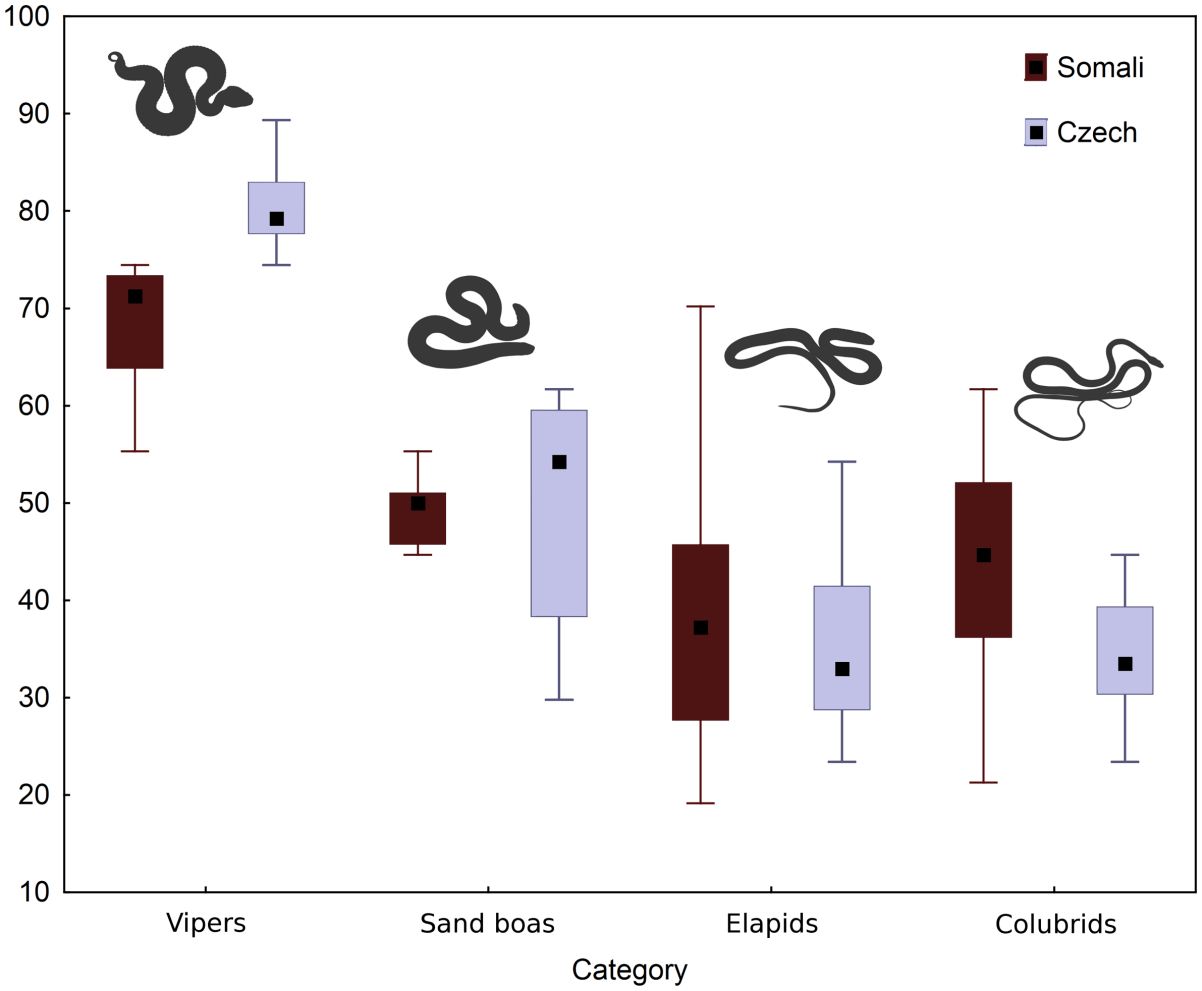
The fear evoked by four categories of snakes in Somali and Czech respondents. Means, quartiles and ranges are provided. The fear index was computed from median values (see under the Material and Methods).

The distribution of subjectively perceived fear among the four *a priori*-defined groups of snake stimuli is visualized in Figure 3. Kruskall-Wallis test revealed a strong effect of the snake group on elicited fear (Somali: H = 25.89; Czech: H = 31.75, both *p* < 0.0001). Within both data subsets, the subjective fear scores of vipers were significantly higher than those of sand boas (Somali: *z* = 3.22, *p* = 0.0076; Czech: *z* = 2.88, *p* = 0.0239), elapids (Somali: *z* = 4.71, *p* < 0.0001; Czech: *z* = 4.88, *p* < 0.0001) and “colubrids” (Somali: *z* = 3.97, *p* = 0.0004; Czech: *z* = 2.83, *p* < 0.0001).

### 3.5. Clustering species according to correlated fear ranks

The tree extracted from fear ranks provided by the Somali respondents has two main branches, each comprising 24 snake species. One contains 11 vipers, 6 sand-boas, 5 elapids and two “colubrids.” The other one, just 1 viper, 6 sand-boas, 7 elapids and 10 “colubrids” (see Supplementary Figure 1).

The tree extracted from the Czech dataset reflects our groups of species more closely. The main branching of the tree corresponds almost precisely to a split between vipers + sand-boas, and elapids + “colubrids.” While elapids and “colubrids” are fairly intermixed within the latter branch, the former one further splits into two distinct branches. One of them includes all vipers, while the other one all sand boas. The position of *Naja mosambica,* belonging to elapids, within the clade of the sand-boas, represents the only violation of this clear pattern (see Supplementary Figure 2).

### 3.6. Correlates of the fear

We calculated Pearson Product–Moment correlation coefficients between fear and visceral traits of the stimuli photographs. The results showed that body width is a good predictor of fear, this relationship we found in both the Somali (*r* = 0.799, df = 47) and the Czech (*r* = 0.815, df = 47) datasets (see Table 1; Supplementary Figures 3, 4).

**Table 1:** Spearman coefficients of correlation between the fear index and 9 measurements of the stimuli.

	Somali		Czech	
	Spearman *r*	*p*-value	Spearman *r*	*p*-value
Total length	−0.1919	0.1915	−0.4018	0.0046
Head length	0.5709	<0.0001	0.3618	0.0115
Head width	**0.7278**	**<0.0001**	**0.6335**	**<0.0001**
Neck width	0.5231	0.0001	0.4846	0.0005
Body width	**0.7994**	**<0.0001**	**0.815**	**<0.0001**
Tail width	0.5001	0.0003	0.3843	0.007
Eye diameter	0.2306	0.1148	−0.0166	0.9108
Perimeter	−0.4726	0.0007	−0.4287	0.0024
Body area	**0.716**	**<0.0001**	**0.6047**	**<0.0001**

The values were calculated separately for Somali and Czech datasets.

### 3.7. Cross-cultural agreement

**Figure 4:**
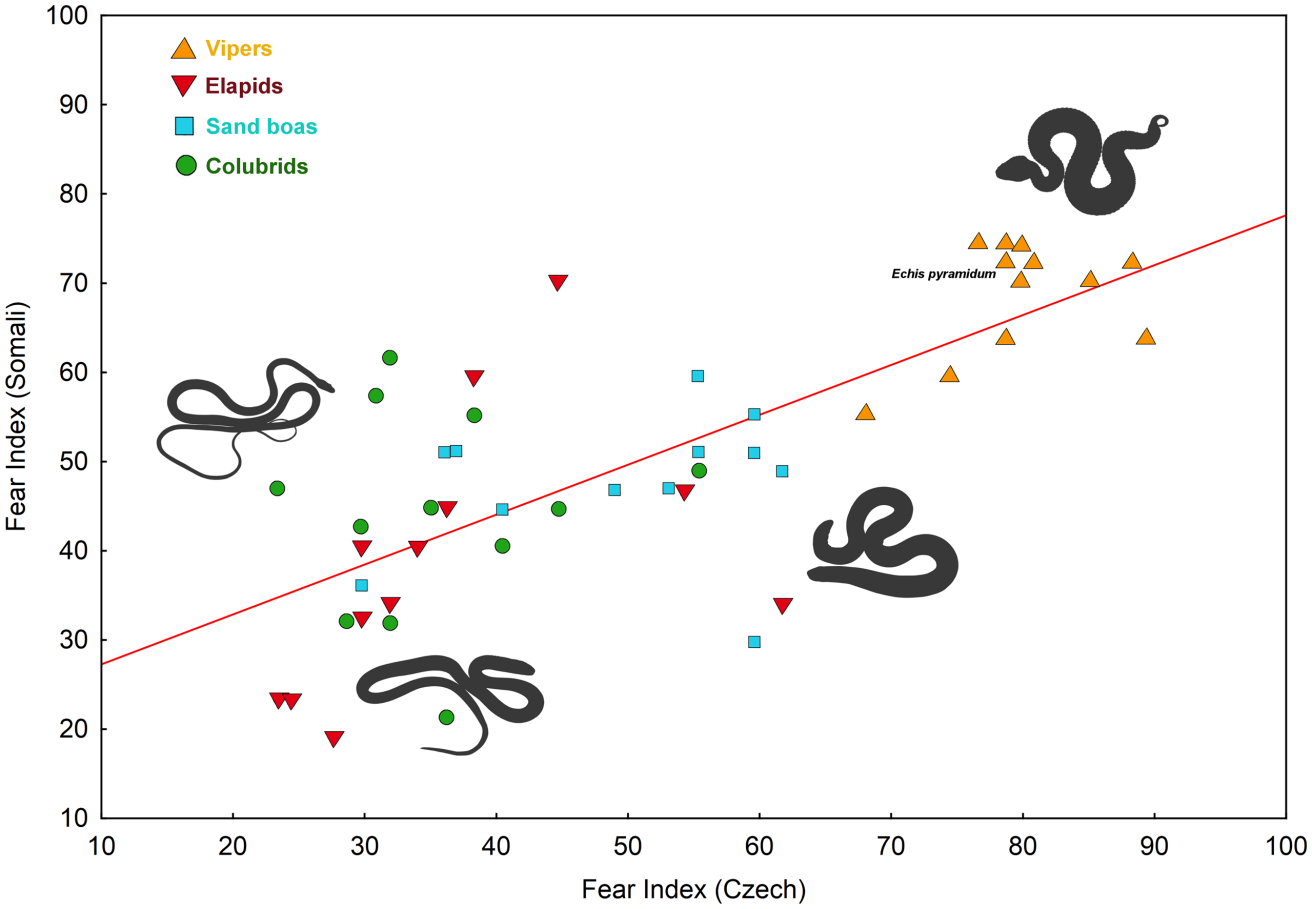
Cross-cultural agreement in the fear evaluation of 48 snake stimuli (Pearson correlation coefficient: *r* = 0.738, *p* < 0.0001). The fear index was computed from median values (see under the Material and Methods).

We detected a considerable correlation between fear indices (see under the Materials and Methods) of the examined snake stimuli assessed in the Somali and the Czech respondents (Pearson Product–Moment *r* = 0.738, df = 47, *p* < 0.0001, see Figure 4). The cross-cultural agreement is, however, probably mediated by the shape of the snake stimulus. This agreement disappeared when the effect of the Body Width of the stimuli was removed by the inclusion of this predictor into the linear model (*F*_(1,45)_ = 3.518, *p* = 0.067).

## 4. Discussion

Our first aim was to assess whether participants exhibited more fear of deadly venomous snakes than of non-venomous or slightly venomous ones. The question of whether humans can distinguish between venomous and non-venomous snakes based on the level of subjectively perceived fear elicited by particular species had not been tested directly, although there have been indications alluding to this question in prior studies (Landová et al., 2018a,b; Janovcová et al., 2019; Rádlová et al., 2019). The selected stimuli were chosen to fit into two groups: venomous snakes represented by the vipers and elapids, and non-venomous snakes represented by the sand boas and “colubrids.” However, this division was not reflected in the relative subjective fear the snakes elicited. Although both vipers and mambas/cobras (elapids) are life-threatening venomous snakes, only the vipers were consistently ranked among the most fear-eliciting stimuli. Contrarily, the vast majority of elapids were placed in the lower half of the scale. We found this important pattern of ranking in both Somalis and Czechs.

### 4.1. The contrasting ranking of viperids and elapids

The discrepancy between the subjective-fear ranking of vipers and elapids contradicts the null hypothesis that all categories of snakes elicit comparable magnitudes of perceived subjective fear. It is reasonable to assume that the sources of selection for quick detection and appropriate behavioral response (mediated by emotional response) were of a similar propensity elicited by both these groups of venomous snakes, at least within the Somali population. Thus, both mambas/cobras and vipers should have been subjectively ranked among the most fear-eliciting snakes. Our data, therefore, do not support the evolutionary-gained specific adaptive emotional response uniform to all venomous snakes. The most important point here is that both Czech and Somali respondents evaluated viperids as the most dangerous snakes according to the subjectively perceived fear. The probable explanation for this phenomenon is the *shared* evolutionary history in Africa, where vipers have posed a serious risk of envenomation. This explanation is compatible with an innate recognition mechanism for the viperid morphotype. An alternative explanation might be that the participants’ ranking reflects an individually learned response, either influenced by personal experiences (shaped by current envenomation risk) or local culture. This alternative also explains well the position of vipers since they are the most (or among the most) dangerous snakes in the home regions of all participants. The low ranking of elapids by Somali participants (except the Mozambique spitting cobra), however, remains puzzling.

What, then, lies behind the relatively higher fear consistently elicited by vipers? We hypothesize that, unlike many other venomous species, vipers are easily recognizable among other snakes and that in this sense, the viperid morphotype is very conspicuous. Several visceral features characterize vipers as a group. Firstly, vipers have a rather short but thick and robust bodies. They have a well-defined triangular head that is separated from the rest of the body by a thinner neck. The majority of viperid snake heads are reminiscent of a pear-shaped arrowhead, featuring relatively sharp angles. Bar and Neta (2006) showed that people perceive objects with sharp-angled contours as potentially more threatening than objects with curved features. This may be another low-level perceptual feature for conscious as well as non-conscious identification of viperids as a potential threat. Their relatively large eyes are prominent and are often accentuated by modified scales. Secondly, the contrasting pattern of dark spots or lines on a light grey or beige background is often present. Thirdly, vipers have large and prominent scales all over the body (the importance of scales for snake detection and recognition was shown by, e.g., Isbell and Etting, 2017; Van Strien and Isbell, 2017; Kawai, 2019; Coss and Charles, 2021). Consequently, vipers appear to have a rugged texture, and their contrasting color pattern (when present) is emphasized. Moreover, all species within the viperid family exhibit a relatively uniform appearance, which facilitates their visual categorization. In essence, vipers possess a distinct morphotype that is conspicuously different from other snakes. It might be that the presence of easily recognizable features is the key factor for forming and fixating the association between these dangerous snakes and the fear response.

### 4.2. The effect of snake morphotype

To further investigate whether a snake’s appearance is associated with its fear ranking, we focused on the analysis of some basic morphological features. Out of all measured parameters, the snake’s body width is the most highly correlated with its fear ranking (Table 1). We also found a moderately high correlation with head width and body area. Moreover, the total body length and body perimeter negatively correlated with perceived fear which means that longer snakes were ranked as relatively less fear-eliciting than shorter snakes. Although these morphological parameters are principally intercorrelated, this result points toward the importance of the snake’s robustness for its emotional evaluation. In our sample, vipers and sand boas represent the robust morphotype with shorter and thicker bodies. As discussed above, the vipers were indeed consistently placed among the most fear-eliciting stimuli and sand boas (when examined as a group) were the second most fear-eliciting (see Figure 3; Supplementary Table S4). Nonetheless, there are some other features that are shared by most vipers and sand boas but absent in most examined elapids and “colubrids.” The most important one is the presence of a scale pattern in contrast to a uniform coloration. It might be that the “conspicuous” scale pattern, not the snake robustness, is the key feature factored by the participants. This question should be addressed in future research.

### 4.3. The differences in ranking of Somalis and Czechs

Our second aim was to compare the subjective-fear ranking of Somali and Czech participants. We found that the cross-cultural agreement on the ranking of all 48 species was 0.738 (df = 47, r^2^ = 0.545) which is relatively high. In our previous work, we compared fear elicited by European and Middle Eastern snakes in Azerbaijani and Czech populations. The cross-cultural agreement on the ranking of these 37 species was 0.826 (df = 36, *r*^2^ = 0.683; Landová et al., 2018a). The higher cross-cultural agreement can be likely attributed to the closer mutual relationship between Czech and Azerbaijani populations, as opposed to Czech and Somali populations, in terms of their population ancestry, culture, and local ophiofauna. Notably, the shape of the snake also played a pivotal role in this study – slender-bodied snakes (colubrids and a cobra in resting position) elicited lower fear than vipers in both Azerbaijanis and Czechs (Landová et al., 2018a). In a different study comparing the ranking of snake beauty among eight populations from five continents, the cross-cultural agreement varied from 0.493 to 0.901 depending on the compared populations (Marešová et al., 2009a; Frynta et al., 2011). Our current result falls within this range. Regarding the attitude toward snakes, we previously identified a more negative attitude among Azerbaijanis in comparison to what was reported by Czech participants (Landová et al., 2018a). However, another study assessing various aspects of attitudes toward snakes among Slovak (also Central Europeans) and Turkish students found no substantial differences in negative attitudes toward snakes, even though these populations differ in diversity and presence of venomous snakes (Prokop et al., 2009).

Nonetheless, an interesting cross-cultural difference comes from the results of cluster analyses. The analyses revealed that vipers formed a relatively distinct cluster separate from other snakes in Czechs and also Somalis. This suggests that the viper stimuli were truly perceived as members of a group and that the group membership (i.e., if the stimulus fits or does not fit into the “viper category”) noticeably affected the species ranking. Contrarily, elapid and “colubrid” snakes got intermixed and did not form any interpretable clusters in either Somalis or Czechs. This shows that the snakes of both groups were perceived as one and that neither Somali nor Czech participants differentiated between them with regard to the elicited fear. Note that all these snakes – both relatively harmless “colubrids” and highly dangerous elapids – were generally ranked below the median. Finally, the sand boas appeared to form its own category only in Czechs; in Somalis, the species were split between the two main clusters. This suggests that in Somalis, the sand boas were evaluated on an individual basis taking into consideration characteristics that do not define sand boas as a group. In fact, the overall structure of the cluster tree was less interpretable in Somalis suggesting that Somali participants took more of an “individual approach” to each snake’s evaluation while Czech participants tended to rank the species based on the group they presumably belong to. Since Somali participants have at least some personal experiences with the stimuli species, they might have evaluated them differently, while Czechs had to rely more on categorization when confronted with these exotic snake stimuli. Alternatively, Czechs might be simply more used to categorizing since semantic categories are ubiquitous during their school education. These two explanations are not mutually exclusive.

### 4.4. The effect of evolutionary past, current snakebite risk, and culture on species’ fear ranking

We outlined three factors that might affect the subjective-fear ranking of the venomous and non-venomous snakes: evolutionary past (i.e., the evolutionary sympatry with dangerous snakes), the current risk of snakebite, and cultural influences (e.g., myths, passed down experiences, media portrayal, education). Nevertheless, none of these three factors on its own can fully explain the observed pattern of ranking. Instead, it appears to us that these factors are not mutually exclusive and that they have all contributed to the fear ranking to varying degrees in both populations.

As large constrictors like pythons have been regular predators of primates (reviewed in Headland and Greene, 2011; Ribeiro-Júnior et al., 2016), and probably also predators of early hominids (Coss, 2003; Isbell, 2006, 2009), the general fear elicited by snakes should be traced back to this deep evolutionary past. However, unlike pythons, venomous snakes are not significant predators of larger primates, and accidents involving envenomation frequently occur when humans step on a hidden snake (Valenta, 2010). Venomous snakes do not actively pursue apes and humans, and some of them (e.g., Indian cobras, *Naja naja*) display face-like patterns with eyespots on the ventral and dorsal sides of their expanded hoods that alarm intruders and potential predators (Ditmars, 1931; Coss, 1968; Ramakrishnan et al., 2005); nevertheless, overlooking them remains risky. It is therefore important for humans not only to detect a hidden snake but also to accurately estimate the risk of a possible bite. An innate wariness specifically toward venomous snakes could be advantageous in this respect.

While our data do not support the existence of an innate fear response uniform for all venomous snakes, they do support the innateness of a stronger fear response toward vipers. Since vipers are morphologically homogenous within the clade consisting of viperid and rattlesnakes but distinct from most other snake groups, forming and fixating an innate “idea” (possibly prototype) of what a dangerous snake looks like might have been advantageous because it would have led to relatively few false alarms. Cobras and mambas, on the other hand, could be easily confused with mostly harmless colubrids leading to a waste of time because of false-positive misidentification or the risk of injury or death because of false-negative misidentification. It might be argued that humans should have hence evolved a fear response toward all vipers, elapids and “colubrids” since false-negative misidentification is clearly much more serious than a false-positive one. Indeed, this is reflected in predominantly negative attitudes toward all snakes across cultures (e.g., Alves et al., 2014; Pandey et al., 2016; Landová et al., 2018a; Onyishi et al., 2021). In this study, the elapids and “colubrids” were among the least fear-eliciting simply because the task was the stimuli ranking, i.e., we assessed only fear in relational context and not a single fear judgment.

The existence of an innate “prototype” of a dangerous snake might be supported by the high ranking of completely harmless sand boas. They were ranked as the second most fear-eliciting group also by Czech participants even though Czechs do not have an opportunity to encounter them in real life (and have not had it for at least 5,000 years). Their high ranking might be attributed to their relative similarity to vipers, a possible morphological key feature might be the relative body robustness but other options like the presence of a coloration pattern are also possible. Somali respondents ranked some sand boas also relatively high; others however were ranked quite low (the highest-ranked sand boa scored 60, the lowest-ranked scored 30 on the fear index scale). Elapids (scoring 70 and 19, respectively) and “colubrids” (scoring 62 and 21, respectively) were also ranked ambiguously. This “individual approach” toward the snake stimuli contrasted with higher reliance on categorization by Czechs. We interpret this result as Somalis adjusting their rating based on their personal experience or second-handily learned information. However, this interpretation should be explored in a follow-up study focusing on a full range of Somali snake species.

The cultural influence on the fear ranking of examined species cannot be easily measured. The higher ranking of vipers than of sand boas might have been caused by their closer resemblance to the possible innate “prototype” of a dangerous snake or alternatively by the culturally transmitted knowledge that vipers are dangerous. In the Czech Republic, already children at school are taught what Northern viper looks like and that it is venomous. The most lethal Somali species, the Northeast African carpet viper, is well known among the people and Somali participants regularly recognized it among the stimuli. While the highest ranking of vipers in both Somalis and Czechs might be explained solely by their characteristic appearance and the historically uninterrupted interaction between this snake family and tested human populations for the last at least 11 million years, from our experience it seems to us that the culture reassures or even amplifies the specific fear reaction toward them.

### 4.5. Comparison with animal studies

Consistent differences in responses to snakes of various species or morphotypes are not surprising in light of previous studies conducted on non-human primates and rodents. For instance, wild Bonnet macaques (*Macaca radiata*) and moor macaques (*M. maura*) exposed to realistic snake models responded differently to each examined snake species (Ramakrishnan et al., 2005; Hernández Tienda et al., 2021). Colombian white-faced capuchins (*Cebus capuchinus*) failed to differentiate between the venomous neotropical rattlesnake (*Crotalus durissus*) and the non-venomous *boa constrictor* (*Boa constrictor*), yet they distinguished highly patterned boas from an unpatterned harmless snake (Meno et al., 2013a,b; Coss et al., 2019). In contrast to capuchins, rodents such as California ground squirrel (*Otospermophilus beecheyi*) and rock squirrel (*Otospermophilus variegatus*) were able to distinguish their venomous rattlesnake and non-venomous gopher-snake predators (Towers and Coss, 1990; Owings et al., 2001).

It is noteworthy that the California ground squirrels’ ability to distinguish both snake predators has persisted under prolonged relaxed selection for more than 300,000 years, following a predator–prey relationship spanning at least 10 million years (Coss, 1991, 1993, 1999). This persistence is analogous to our findings, given that the evolutionary interaction between human ancestors and venomous snakes in Africa lasted for several million years and fear response to snakes (and specifically to viperids) currently occurs in European populations even after migration to areas where venomous snakes are rare or absent (< 60,000 years ago).

Another parallel to our results can be found in the ability of moor macaques (*M. maura*) to generalize their previous experience with local vipers to a novel viper species (Hernández Tienda et al., 2021). The authors attributed this ability to the triangular shape of viper heads. Further, moor macaques only poorly responded to cobras and kraits (*Bungarus* spp.); they paid the most attention to large constrictors (pythons) regularly preying on macaques (Shine et al., 1998; Headland and Greene, 2011). This aligns with other studies conducted on monkeys, reporting a preference for emitting alarm calls in response to large pythons (Van Schaik and Mitrasetia, 1990; Ramakrishnan et al., 2005; Coss et al., 2007). These alarm calls are not exclusive to pythons and boas (their acoustic characteristics do not possess unique attributes for constrictors). Instead, they reflect the level of threat and are further influenced by the animals’ experiences with encountered predators. They may also serve as highly contagious alerting signals directed at the other members of the group (Crockford et al., 2012; Coss et al., 2019).

Differences in responses to constrictors and venomous snakes, as demonstrated by some studies in non-human primates and other animals, are mostly overlooked in humans. Large ancestral pythons coincided temporally with early hominids 4.5 million years ago and likely posed a predation risk to them, even though no paleontological evidence exists (see Coss, 2003; Headland and Greene, 2011). Our study showed that the subjectively perceived fear of sand boas was lower than that of vipers, but large pythons were not included. However, a previous study comparing subjectively perceived fear revealed that out of 40 randomly selected representatives of extant snake subfamilies, viperids occupied the 1st (Crotalinae), 2nd (Viperinae) and 4th (Azemiopinae) position while subfamilies comprising large constrictors were 6th (Boinae), 17th (Sanziniinae) and 19th (Pythoniinae) (for details see Rádlová et al., 2019, and its Supplementary material 1). Nonetheless, additional research is needed to uncover potential differences in various aspects of human fear reactions to venomous snakes and large constrictors.

## Data availability statement

The original contributions presented in the study are included in the article/Supplementary material, further inquiries can be directed to the corresponding author.

## Ethics statement

The studies involving humans were approved by Institutional Review Board of Charles University, Faculty of Science and Amoud University, School of Postgraduate Studies & Research. The studies were conducted in accordance with the local legislation and institutional requirements. The participants provided their written informed consent to participate in this study.

## Author contributions

DF conceived and designed the research, analyzed the data, and funding acquisition. DF, PF, HE, IŠ, VR, KR, DK, DS, and DB recruited the respondents and administered the tasks in Somaliland. MJ, IŠ, PF, VR, and EL administered the tasks in the Czech Republic. MJ curated the data and prepared the stimuli. DF and PF photographed the stimuli. DF, EL, IŠ, PF, VR, and MJ wrote a first draft of the manuscript. DF, EL, IŠ, and PF reviewed the text. All authors approved the final version of the manuscript.

## Funding

This study was supported exclusively by the Czech Science Foundation (no. GACR 20-21608S) awarded to DF.

## Conflict of interest

The authors declare that the research was conducted in the absence of any commercial or financial relationships that could be construed as a potential conflict of interest.

## Publisher’s note

All claims expressed in this article are solely those of the authors and do not necessarily represent those of their affiliated organizations, or those of the publisher, the editors and the reviewers. Any product that may be evaluated in this article, or claim that may be made by its manufacturer, is not guaranteed or endorsed by the publisher.
