# Supplementary material for: Are vipers prototypic fear-evoking snakes? A cross-cultural comparison of Somalis and Czechs
Source: Front Psychol. 2023 Oct 19;14:1233667. doi: 10.3389/fpsyg.2023.1233667 (PMC10620321; doi:10.3389/fpsyg.2023.1233667)
Supplement: Supplementary file 2 [file Table_2.pdf]

frontiers

Supplementary Material

Are vipers prototypic fear-evoking snakes? A cross-cultural comparison of Somalis and Czechs

Daniel Frynta[1], Hassan Sh Abdirahman Elmi[2,1], Markéta Janovcová[1], Veronika Rudolfová[1], Iveta Štolhoferová[1], Kateřina Rexová[1], David Král[1], David Sommer[1], Daniel Alex Berti[1], Eva Landová[1], Petra Frýdlová[1*]

*** Correspondence:** Petra Frýdlová: petra.frydlova@natur.cuni.cz

Supplementary Figures

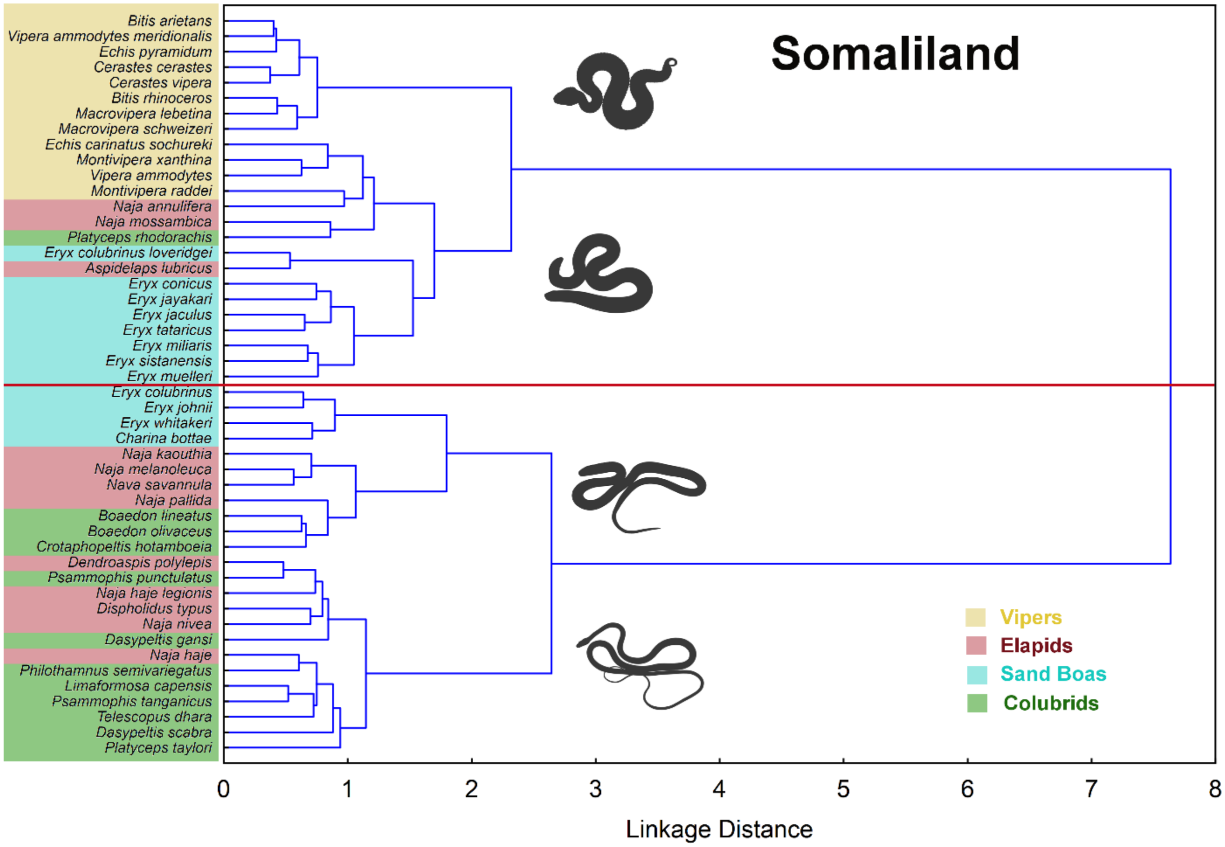

Supplementary Figure 1. Results of the cluster analysis of the Somali dataset. We extracted the dissimilarity matrix from the ranking dataset (1-Pearson's r) and applied Ward's method of clustering.

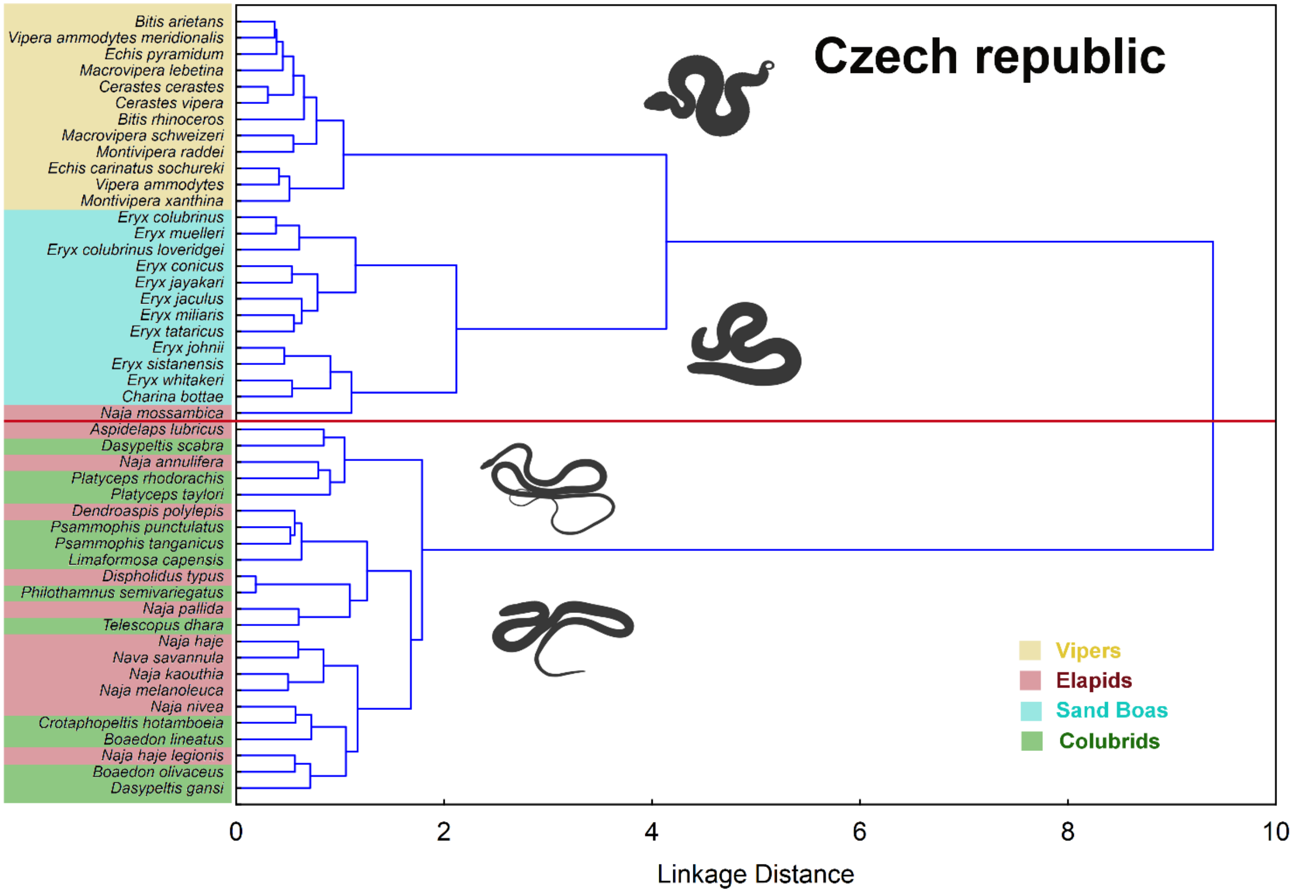

Supplementary Figure 2. Results of the cluster analysis of the Czech dataset. We extracted the dissimilarity matrix from the ranking dataset (1-Pearson’s r) and applied Ward’s method of clustering.

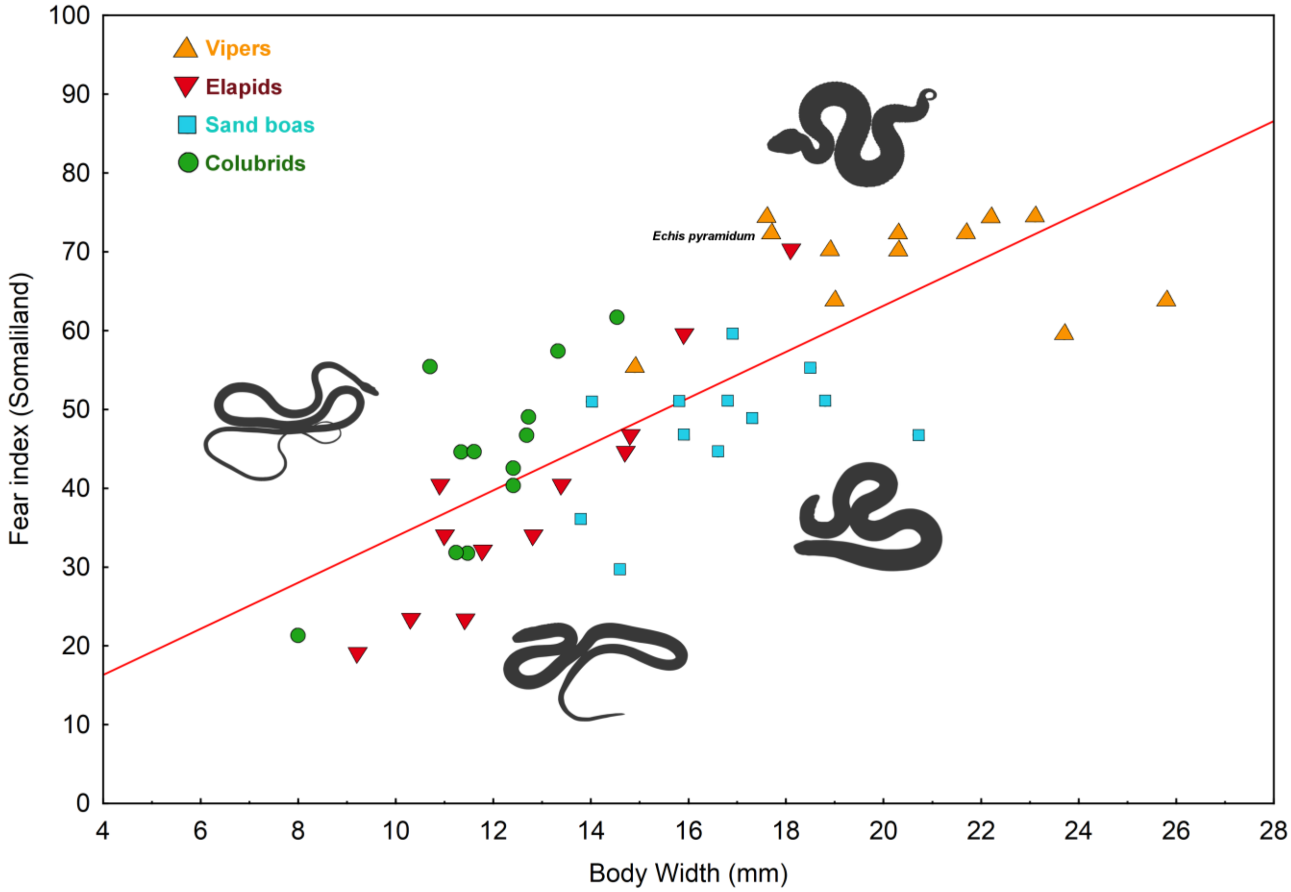

Supplementary Figure 3. A scatter plot of the fear index extracted from the Somali dataset against the Body Width of the stimulus. Pearson's correlation coefficient r = 0.783.

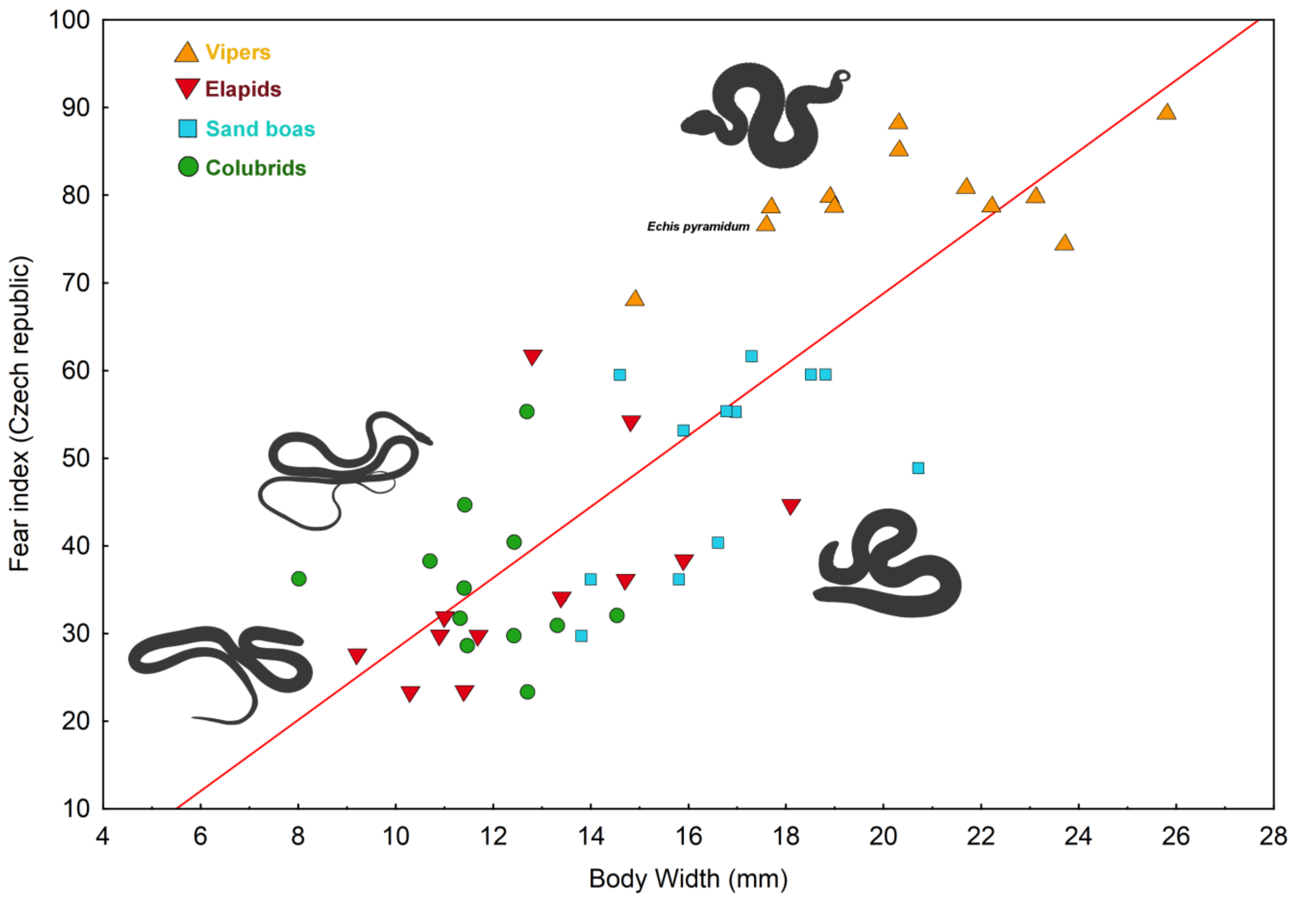

Supplementary Figure 4. A scatter plot of the fear index extracted from the Czech dataset against the Body Width of the stimulus. Pearson's correlation coefficient r = 0.821.
